# Integration of single-cell and bulk RNA sequencing reveals divergent T-cell immune circuits and a shared osteo-immune pathway in spinal tuberculosis and brucellar spondylitis

**DOI:** 10.3389/fcimb.2026.1811708

**Published:** 2026-05-19

**Authors:** Zongqiang Yang, Xuefeng Yue, Qiang Liu, Zhangui Gu, Long Ma, Zhenyong Tao, Kun Wang, Daihao Wei, Rui Huang, Jin Hu, Ningkui Niu, Jiandang Shi

**Affiliations:** 1 Department of Orthopaedic, General Hospital of Ningxia Medical University, Yinchuan, Ningxia Hui Autonomous Region, China; 2 Ningxia Medical University, Yinchuan, Ningxia Hui Autonomous Region, China; 3 Research Centre for Prevention and Control of Bone and Joint Tuberculosis, General Hospital of Ningxia Medical University, Yinchuan, Ningxia Hui Autonomous Region, China; 4 Preparation Centre, General Hospital of Ningxia Medical University, Yinchuan, Ningxia Hui Autonomous Region, China

**Keywords:** Spinal tuberculosis, Brucella spondylitis, single-cell RNA sequencing, T-cell immune landscape, osteo-immune axis

## Abstract

**Background:**

Spinal tuberculosis (STB) and Brucella spondylitis (BS) are the two most common infectious diseases of the spine. Despite overlapping clinical features, their immunopathological mechanisms remain poorly defined.

**Methods:**

We performed integrated transcriptomic profiling of infected nucleus pulposus tissues from STB and BS patients, combining single-cell RNA sequencing (STB n=3; BS n=3) with bulk RNA sequencing (STB n=7; BS n=5). Single-cell analysis resolved immune heterogeneity and T-cell subset architecture, whereas bulk RNA-seq defined global transcriptional signatures. Cross-platform integration identified concordant differentially expressed genes and shared pathways. AUCell was applied to quantify T-cell functional programmes. Pseudotime trajectory reconstruction, regulon inference, and cell-cell communication analyses were performed to delineate immune circuit differences.

**Results:**

A total of 67,274 high-quality cells were analysed, identifying nine major cell populations, with T cells as a dominant immune component. STB tissues were enriched in inflammatory cells, including monocytes/macrophages, neutrophils, and osteoclasts, whereas BS showed higher proportions of B and T cells. In STB, CD4 T cells were skewed toward IL26-CD4 T, Cytotoxic CD4 T, Proliferating CD4 T, and Treg subsets, while CD8 T cells were enriched in IFNG-AS1-CD8 T and exhaustion-like populations, reflecting sustained IFN-driven activation with proliferative and apoptotic stress. In contrast, BS exhibited expansion of GZMB-CTLs and IFNG-CTLs subsets, consistent with a focused cytotoxic programme with preserved memory features. Integrated single-cell and bulk analyses demonstrated convergence of overlapping genes on TCR, NF-κB, cytokine signalling, and osteoclast differentiation pathways. Functional scoring and pseudotime analysis indicated broader Th1/Th17 activation and progression toward terminal exhaustion in STB, whereas BS T cells maintained intermediate effector–memory states. Regulatory network analysis revealed a STAT1/IRF-dominated interferon axis in STB and an IRF4/EOMES/RFX3-centred effector–memory programme in BS. Cell-cell communication mapping showed an MHC-II-CD4 axis predominating in STB and an MHC-I-CD8 axis in BS. Despite divergent immune circuits, both conditions converged on osteoclast differentiation pathways, suggesting immune–bone metabolic coupling as a shared mechanism of vertebral destruction.

**Conclusions:**

STB and BS exhibit pathogen-specific T-cell immune circuits that converge on a common osteo-immune pathway, providing mechanistic insight and potential targets for host-directed therapy.

## 1 Introduction

Infectious diseases of the spine (IDS) are infections of the vertebral bodies, intervertebral discs, posterior elements, the spinal canal, and adjacent paraspinal tissues caused by diverse pathogenic microorganisms, and they account for approximately 2–7% of musculoskeletal infections (Tsantes et al., 2020; Liu et al., 2021). Spinal tuberculosis (STB) and Brucella spondylitis (BS) are the most common IDS. In recent years, the persistence of zoonotic diseases in pastoral regions, the resurgence of tuberculosis, rising antimicrobial resistance, and an expanding immunocompromised population have driven a sustained increase in the incidence of BS and STB, particularly in northwestern China (Wen et al., 2024; Cao et al., 2025). These conditions have consequently emerged as major public-health concerns, imposing a substantial burden on patients and healthcare systems (Li et al., 2023; Lu et al., 2024; Cao et al., 2025). Both BS and STB usually present with systemic symptoms such as fever, night sweats, fatigue, and weight loss, together with local signs including pain, restricted mobility, and neurological deficits, however, these manifestations are non-specific and frequently overlap (Hammami et al., 2021; Yasin et al., 2023). Radiological features of both conditions commonly include intervertebral space narrowing and vertebral body destruction. STB is more often characterised by extensive vertebral body destruction, paravertebral or epidural abscess formation, and kyphotic deformity, whereas BS typically involves endplate lesions and focal changes. These distinctions have been confirmed by multiple comparative studies and MRI-based radiomics analyses (Sharif et al., 1989; Liu et al., 2018; Wang et al., 2023b). Nonetheless, clinical and imaging features alone are insufficient for definitive differentiation, and in endemic regions, misdiagnosis and diagnostic delay remain common.

Although pathogen detection remains the gold standard for definitive diagnosis, its practical clinical application is often limited. In human brucellosis, the positivity of blood culture is closely related to disease stage, with higher positivity in the acute phase and a marked decline in chronic disease. A large cohort study stratified by stage reported positivity rates of 35.9% in acute cases, 11.1% in subacute cases, and only 1.9% in chronic cases, underscoring the difficulty of confirming chronic manifestations such as spinal involvement through blood culture alone (Yagupsky et al., 2019). In spinal tuberculosis, conventional Lowenstein–Jensen culture requires 6–8 weeks with a sensitivity of approximately 47%, whereas liquid culture systems such as MGIT can reduce the time to 4–10 days but achieve only about 56% sensitivity, indicating limited overall diagnostic efficiency (Colmenero et al., 2013; Shanmuganathan et al., 2023). Consequently, biopsy, molecular assays, and histopathological evaluation are frequently required to establish a reliable diagnosis.

Previous studies have demonstrated that T cells are key regulators of the local immune response in infectious spondylitis. In tuberculosis, CD4 T cells play a central role by secreting IFN-γ through Th1-mediated pathways to activate macrophages, while Th17 cells contribute to inflammation and neutrophil recruitment, thereby supporting granuloma integrity and sustaining immune defence. In parallel, CD8 T cells exert cytotoxic activity via perforin- and granzyme-mediated mechanisms, directly eliminating infected cells (Mpande et al., 2021; Vats et al., 2024). Furthermore, the persistence and reactivation of memory T cells are critical for maintaining long-term immune control during chronic tuberculosis infection (Liu et al., 2023). By contrast, immunological studies of brucellosis indicate that T cell responses are frequently modulated or suppressed by the pathogen. Reviews have shown that patients with Brucella infection often exhibit cellular hyporesponsiveness and impaired Th1 function, thereby compromising effective clearance (Skendros et al., 2011, 2007). *Brucella abortus* has been reported to attenuate T cell activity through PD-L1–mediated inhibitory signalling, leading to immune exhaustion and hyporesponsiveness (Pellegrini et al., 2025a). Therefore, systematic comparison of T cell lineage composition, functional potential, and transcriptional regulation within local lesions of BS and STB may fill critical gaps in clinical diagnosis and management. Theoretically, such insights could also provide a foundation for precision immunomodulation and targeted therapeutic strategies.

Single-cell sequencing is a transformative technology that enables high-resolution analysis of genomic and transcriptomic profiles in individual cells (Wilk et al., 2020), it has been utilized to explore the heterogeneity of immune cells within tuberculous lesions (Wang et al., 2023a). However, systematic investigations directly comparing T cell lineage composition, functional potential, and transcriptional regulation within local lesions of STB and BS remain scarce, representing a critical knowledge gap in both clinical diagnosis and personalised therapy. By integrating single-cell sequencing with bulk transcriptomics, the present study aims to generate a comprehensive immune atlas of T cell responses to STB and BS, and to elucidate the immunological differences between the two diseases. Specifically, this work seeks to characterise lineage composition, functional states, differentiation trajectories, transcriptional regulatory networks, and intercellular communication patterns of T cells within local lesions. This approach provides a feasible strategy to elucidate the immunological mechanisms underlying STB and BS, supports differential diagnosis, and may guide the development of targeted therapeutic interventions.

## 2 Materials and methods

### 2.1 Inclusion and exclusion criteria

#### 2.1.1 Inclusion and exclusion criteria

Inclusion criteria: (1) Patients diagnosed with STB based on clinical history, clinical manifestations, laboratory investigations, and imaging findings, further confirmed by the detection of Mycobacterium tuberculosis in puncture or surgically resected tissue specimens using conventional bacterial culture, Xpert MTB/RIF, or mNGS/tNGS (Tao et al., 2025), or by histopathological evidence of acid-fast bacilli positivity. (2) Patients diagnosed with BS based on clinical history, clinical manifestations, laboratory investigations, and imaging findings, further confirmed by the detection of Brucella species in puncture or surgically resected specimens using conventional bacterial culture or mNGS/tNGS, a positive standard tube agglutination test (≥1:160), or a positive blood culture for Brucella. (3) Clear surgical indications. (4) Adequate quantity and quality of tissue specimens obtained. (5) Good patient compliance and willingness to participate.

#### 2.1.2 Exclusion criteria

(1) Concurrent tuberculosis or brucellosis affecting other organ systems. (2) Presence of systemic autoimmune or immunodeficiency disorders. (3) Poor general condition or severe systemic comorbidities. (4) Contraindications to surgical intervention.

### 2.2 Clinical data and sample collection

#### 2.2.1 Clinical data

According to the inclusion and exclusion criteria, a total of 18 patients diagnosed with either BS or STB who underwent surgical treatment in our department, were included in this study. The clinical characteristics of all patients are summarised in **Supplementary Table 1** (Supporting Information). Written informed consent was obtained from all participants prior to enrolment, and the study protocol was reviewed and approved by the Ethics Committee.

#### 2.2.2 Sample collection

All patients underwent surgery under general anaesthesia via combined anterior-posterior, anterior-only, or posterior-only approaches. Surgical management included thorough debridement of infected lesions, intervertebral bone graft fusion, and internal fixation. Intervertebral disc specimens were obtained from three patients with STB and three with BS according to the inclusion and exclusion criteria. To ensure high cell viability for single-cell sequencing, tissue freshness was strictly preserved. Immediately following debridement, lesion tissues were placed in chilled saline and gently rinsed. Under sterile conditions, samples were washed twice with pre-cooled RPMI-1640 medium containing 0.04% BSA, transferred into preservation solution, and maintained at 4 °C. Samples were transported in temperature-controlled containers to OE Biotech Co., Ltd. (Shanghai, China) within 12 h. Care was taken throughout handling and transport to minimize mechanical and thermal stress, reduce ex vivo time, and preserve cellular integrity. For bulk RNA sequencing, a portion of each specimen was incubated overnight at 4 °C in RNA stabilization reagent and stored at -80 °C.

### 2.3 Single-cell RNA sequencing and data analysis

#### 2.3.1 Preparation and quality control of single-cell suspensions

Under sterile conditions, tissues were washed twice with pre-cooled RPMI-1640 medium containing 0.04% BSA. Samples were minced into ~0.5 mm³ fragments using sterile scissors and transferred to freshly prepared enzymatic digestion solution. Digestion was carried out at 37 °C for 30–60 min with gentle inversion every 5–10 min to promote dissociation. The cell suspension was filtered through a 40 μm BD cell strainer (once or twice) and centrifuged at 300 × g for 5 min at 4 °C. The pellet was resuspended in medium and treated with an equal volume of red blood cell lysis buffer (MACS, Cat. no. 130-094-183), followed by incubation at 4 °C for 10 min. After centrifugation at 300 × g for 5 min, the supernatant was discarded. Cells were washed once with fresh medium, centrifuged, and resuspended in 100 μL of medium. Cell concentration and viability were determined using a Luna™ automated cell counter.

#### 2.3.2 Library preparation and sequencing

Single-cell suspensions were assessed for viability, and only samples with >90% viability were used for library preparation. Cell suspensions were adjusted to a final concentration of 700-1,200 cells/μL in accordance with the manufacturer’s recommendations. Library preparation was performed using the Chromium Next GEM Single Cell 3′ Reagent Kits v3.1 (10x Genomics, Cat. No. 1000268). The resulting libraries were sequenced on the Illumina NovaSeq 6000 platform with a paired-end 150 bp (PE150) configuration.

#### 2.3.3 Gene quantification and quality control

Raw sequencing data were processed using Cell Ranger (10x Genomics, USA), which utilizes the STAR aligner (Dobin et al., 2013)for read alignment to the reference genome. Standard quality control metrics, including detected gene counts, unique molecular identifier (UMI) counts, and mapping rates, were generated for initial data assessment.

Quality control filtering was subsequently performed to exclude low-quality cells, doublets, and empty droplets. Cells were removed if they exhibited fewer than 200 detected genes, <1,000 UMIs, log10(genes per UMI) < 0.7, mitochondrial UMI content >10%, or haemoglobin gene content >5%. DoubletFinder (McGinnis et al., 2019) was further applied to identify and remove residual doublets. The remaining high-quality single cells were retained for subsequent analyses.

#### 2.3.4 Dimensionality reduction and clustering analysis

To correct for batch effects across samples, data integration was performed using the mutual nearest neighbours (MNN) algorithm. Following MNN correction, low-dimensional embeddings were generated and visualized using uniform manifold approximation and projection (UMAP). Cell clustering was conducted using a graph-based method based on the shared nearest neighbour (SNN) algorithm, enabling the identification of transcriptionally distinct cell populations.

#### 2.3.5 Differential gene expression and enrichment analysis

Differentially expressed genes (DEGs) were identified using the FindMarkers function in the Seurat package with the presto method (test.use = “presto”). Genes with an adjusted p value (Benjamini-Hochberg correction) < 0.05 and a fold change > 1.5 were considered statistically significant. Functional enrichment analysis was performed using Gene Ontology (GO) and Kyoto Encyclopaedia of Genes and Genomes (KEGG) pathway analyses. Statistical significance was assessed using the hypergeometric test.

#### 2.3.6 Pseudotime trajectory analysis

Pseudotime trajectory analysis was performed using the Monocle package (Trapnell et al., 2014), which applies machine learning algorithms to reconstruct dynamic cellular transitions during differentiation based on key gene expression patterns. Genes showing high expression variability across cells were first selected, and dimensionality reduction was then applied to their expression profiles. Subsequently, a minimum spanning tree (MST) was constructed, with the longest path within the MST representing the inferred differentiation trajectory of transcriptionally similar cells.

#### 2.3.7 Regulon analysis

Regulon activity was inferred using the SCENIC package (Van De Sande et al., 2020), which reconstructs gene regulatory networks by identifying transcription factor (TF)-driven co-expression modules and their candidate target genes (regulons). Regulon activity at the single-cell level was quantified using the regulon activity score (RAS).

To characterize cell type-specific regulatory programs, the regulon specificity score (RSS) was calculated to evaluate the enrichment of each regulon across distinct cell populations. Furthermore, the connection specificity index (CSI) was employed to quantify the relationships among regulons, with higher CSI values indicating stronger co-regulatory interactions and coordinated functional roles.

#### 2.3.8 Cell-cell communication analysis

Cell-cell ligand-receptor interactions were inferred using the CellChat R package (version 2.1.2) (Jin et al., 2021). Normalized expression matrices were used to construct a CellChat object with the createCellChat function. Preprocessing was conducted under default settings, including identification of overexpressed genes and interactions using identifyOverExpressedGenes and identifyOverExpressedInteractions, followed by data projection with projectData.

Communication probabilities were computed using computeCommunProb and subsequently filtered using filterCommunication (min.cells = 10). Signalling pathway-level communication was inferred with computeCommunProbPathway, and the overall intercellular communication network was aggregated using the aggregateNet function.

### 2.4 Bulk RNA sequencing and data analysis

#### 2.4.1 RNA extraction and library construction

Total RNA was extracted using TRIzol reagent (Invitrogen, USA) according to the manufacturer’s instructions. RNA purity and concentration were determined with a NanoDrop 2000 spectrophotometer (Thermo Scientific, USA), and RNA integrity was assessed using an Agilent 2100 Bioanalyzer (Agilent Technologies, Santa Clara, CA, USA). Transcriptome libraries were constructed using the VAHTS Universal V6 RNA-seq Library Preparation Kit, following the manufacturer’s protocol. Bulk RNA sequencing and subsequent bioinformatic analyses were performed by OE Biotech Co., Ltd. (Shanghai, China).

#### 2.4.2 RNA sequencing and differential gene expression analysis

Libraries were sequenced on an Illumina NovaSeq 6000 platform to generate 150 bp paired-end reads. Raw FASTQ files were processed using fastp (Chen et al., 2018) to trim adapters and remove low-quality reads, producing high-quality clean reads for subsequent analyses. Read alignment was carried out against the reference genome using HISAT2 (Kim et al., 2015), and gene expression was quantified as fragments per kilobase of transcript per million mapped reads (FPKM) (Roberts et al., 2011). Gene-level read counts were obtained using HTSeq-count (Anders et al., 2015).

Principal component analysis (PCA) and data visualisation were performed in R (v3.2.0) to evaluate biological reproducibility and sample clustering. Differentially expressed genes (DEGs) were identified using DESeq2 (Love et al., 2014), with significance thresholds of *p* < 0.05 and fold change > 2 or < 0.5. Finally, hierarchical clustering of DEGs was conducted in R (v3.2.0) to visualise expression patterns across groups and samples.

#### 2.4.3 Integration of bulk and single-cell RNA-seq data

To evaluate the consistency between bulk and single-cell RNA-seq data, we performed deconvolution analysis to infer cell-type composition from bulk RNA-seq profiles. In addition, representative marker genes for major cell types identified in single-cell RNA-seq were used to construct a heatmap based on bulk RNA-seq expression data. Pearson correlation analysis was further conducted to assess the concordance between bulk RNA-seq profiles and single-cell-derived expression signatures.

The raw sequencing data generated in this study have been deposited in the Genome Sequence Archive for Human (GSA-Human) under accession numbers HRA017497 (single-cell RNA-seq) and HRA017547 (bulk RNA-seq).

### 2.5 Statistical analysis

All statistical analyses were performed using R software (v3.2.0) or GraphPad Prism (v9.0), incorporating the appropriate bioinformatics and statistical packages. Unless otherwise stated, two-sided Wilcoxon rank-sum tests were used to determine statistical significance, and results are reported as *p*-values. Comparisons between two independent groups were conducted using unpaired, two-tailed Student’s t-tests. Data are presented as the mean ± standard error of the mean (SEM) or as the median with interquartile range (IQR), depending on data distribution. Statistical significance was defined as follows: ****p < 0.0001; ***p < 0.001; **p < 0.01; *p < 0.05; ns, not significant (*p* > 0.05).

## 3 Results

### 3.1 Single-cell landscape of nucleus pulposus in human subjects with BS and STB

**Figure 1:**
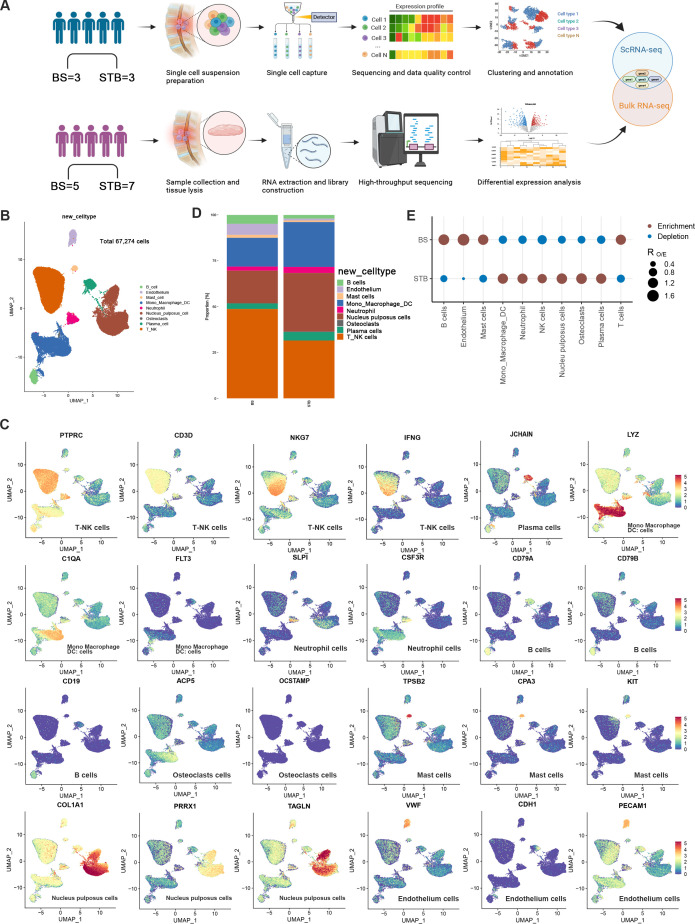
ScRNA-seq of nucleus pulposus tissues from BS and STB patients. **(A)** The over flowchart of the research. **(B)** UMAP visualisation of nine major cell types identified across six samples (n = 67,274 cells after quality control), coloured by cell type (left) and group (right). **(C)** Feature plots showing marker gene expression profiles for nine subsets. **(D)** Comparison of the relative proportions of nine cell populations between BS and STB. **(E)** Ro/e analysis indicating preferential enrichment of major cell populations in BS and STB.

To delineate the cellular architecture of infected intervertebral disc tissues, we performed scRNA-seq on nucleus pulposus samples from three patients with STB and three with BS, and bulk RNA sequencing on an independent cohort comprising seven STB and five BS patients. The overall study design is illustrated in **Figure 1A**. After stringent quality control and batch-effect correction using the mutual nearest neighbours (MNN) algorithm, 67,274 high-quality cells were retained for downstream analysis. Uniform manifold approximation and projection (UMAP) based on MNN-corrected embeddings revealed nine transcriptionally distinct clusters (**Figures 1B, C**). Cell-type annotation based on canonical marker genes and published single-cell datasets identified nine major cell populations: T-NK cells, nucleus pulposus (NP) cells, monocytes, macrophages, and dendritic cells (Mono/Macro/DC), plasma cells, endothelial cells, B cells, neutrophils, mast cells, and osteoclasts (**Figures 1B, C****;** **Supplementary Table 2**). Quantitative analysis of cellular composition demonstrated that T cells, NP cells, and Mono/Macro/DCs constituted the predominant populations in both STB and BS tissues (**Figure 1D**). However, comparative Ro/e enrichment analysis revealed significant disease-specific differences in cellular distribution. STB tissues were markedly enriched in inflammatory-associated cell types, including Mono/Macro/DCs, neutrophils, NK cells, NP cells, osteoclasts, and plasma cells. In contrast, BS tissues exhibited relative enrichment of B cells, endothelial cells, mast cells, and T cells (**Figure 1E**). These findings suggest that, within the present disease-to-disease comparison framework, BS and STB share a broadly similar cellular architecture, while STB is characterised by a more inflammation-dominated microenvironment and BS exhibits a relatively lymphocyte-enriched immune landscape, with a notable contribution from B cells.

To further investigate the relationship between bulk RNA-seq and scRNA-seq data, we performed deconvolution analysis, marker gene heatmap visualisation, and cross-platform correlation analysis (**Supplementary Figures S1A–C**). These results demonstrate that bulk RNA-seq captures tissue-level cellular composition and transcriptional patterns consistent with single-cell findings.

### 3.2 Dissection and clustering of T-NK cells in BS and STB

**Figure 2:**
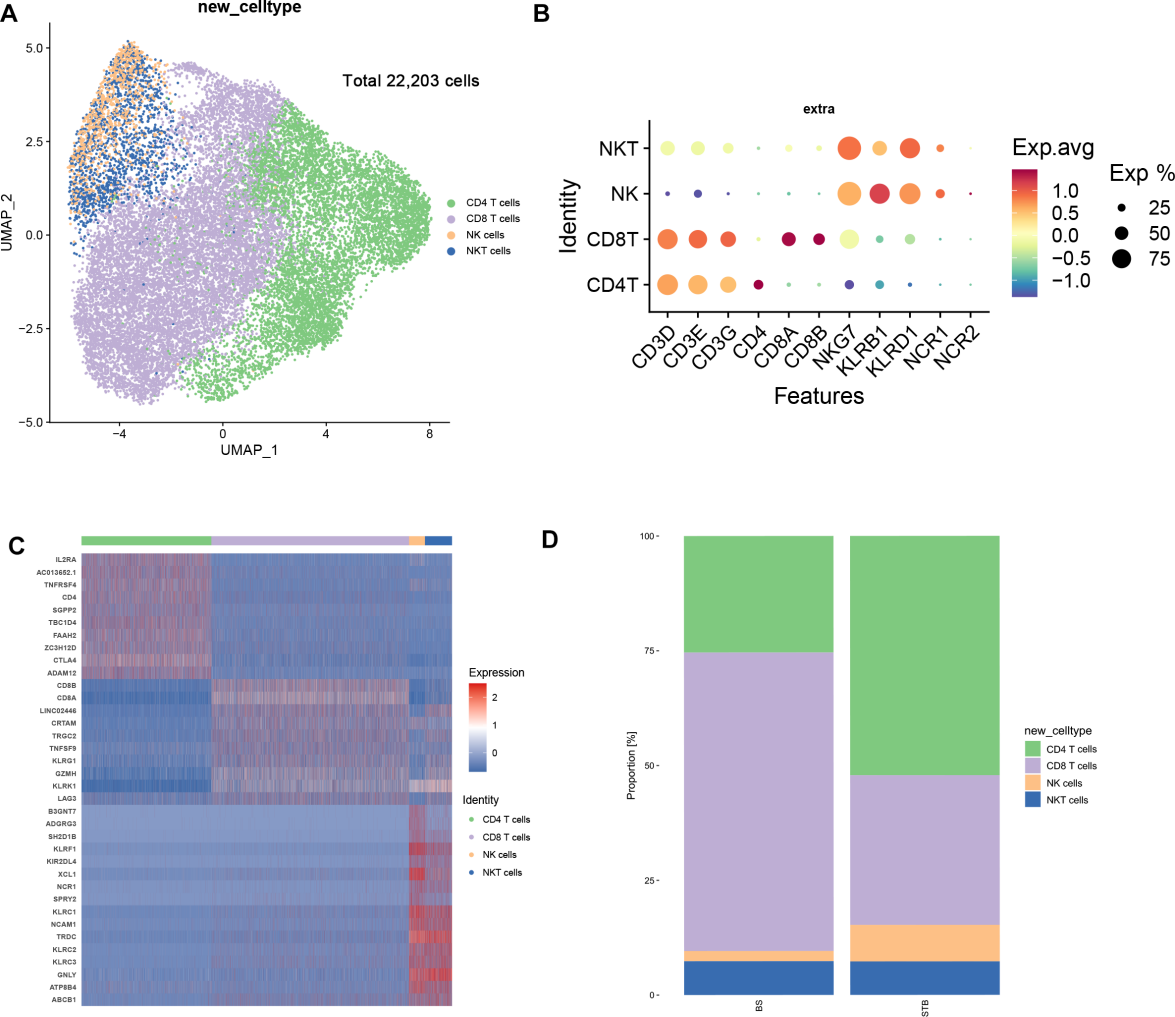
Dissection and clustering of T-NK cells in BS and STB. **(A)** UMAP visualisation of T-NK cell subclusters identified from BS and STB samples (n = 22,203 cells after quality control). **(B)** Dot plot showing the expression levels of canonical marker genes defining each T-NK cell subset. **(C)** Heatmap displaying the top marker genes defining each T-NK subset. **(D)** Proportional distribution of T–NK subsets between BS and STB.

Analysis of immune cells within the nucleus pulposus tissues revealed marked differences in lymphocyte composition between BS and STB, apart from variations in macrophage abundance. To further characterise these lymphocytic differences, all lymphocytes were extracted and reclustered, identifying four major subpopulations: CD4 T cells, CD8 T cells, NK cells, and NKT cells (**Figure 2A**). The relative expression profiles of canonical marker genes used for cell-type annotation are presented in **Figure 2B**, and the top genes defining each subset are shown in **Figure 2C**. Quantitative comparison of these subsets revealed distinct enrichment patterns between the two groups: CD8 T cells and NKT cells were more abundant in BS, whereas CD4 T cells and NK cells were predominantly enriched in STB (**Figure 2D**).

### 3.3 Single-cell and bulk RNA-seq reveal differences in CD4 T cell subsets between BS and STB

**Figure 3:**
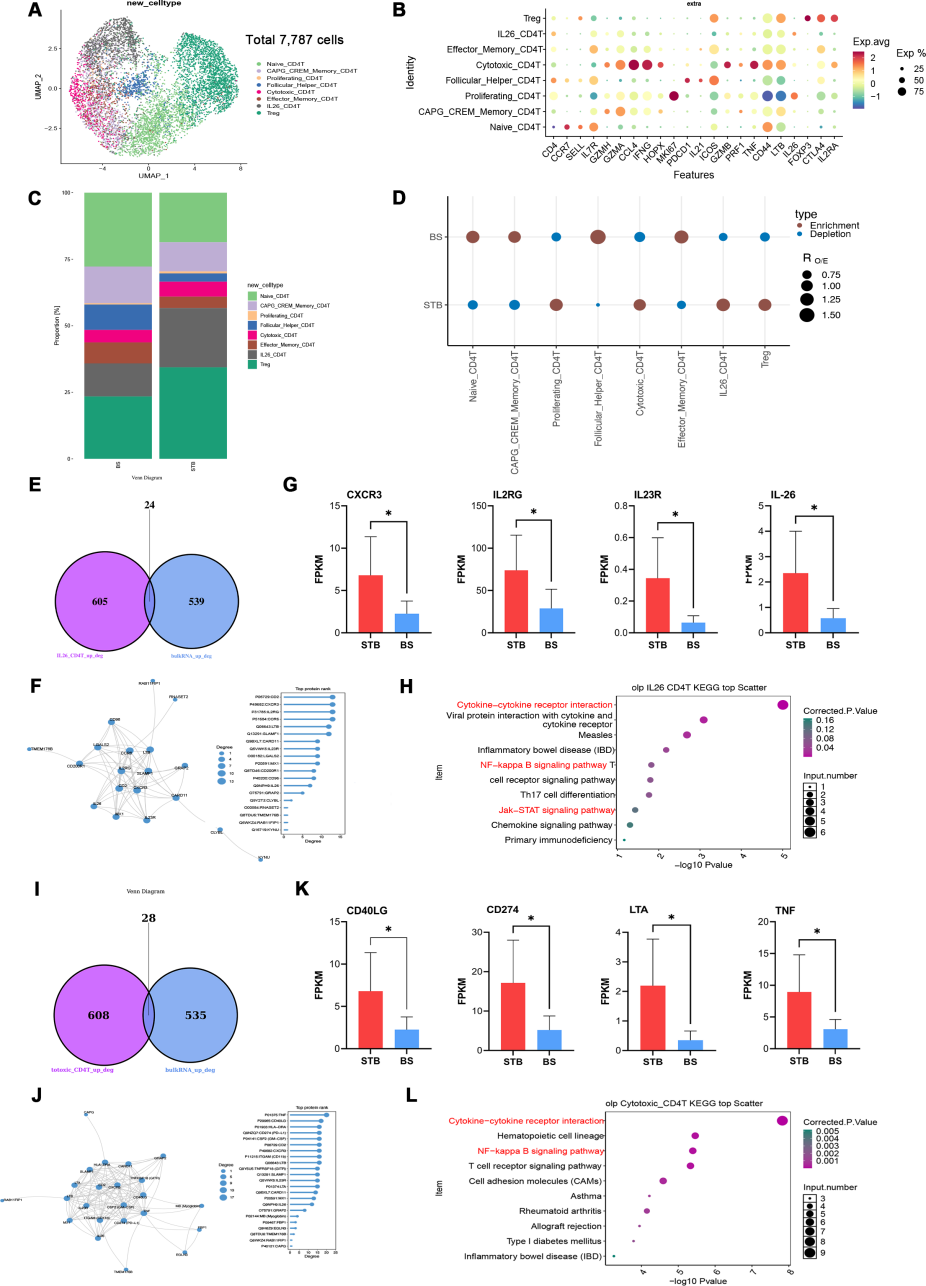
Single-cell and bulk RNA-seq analyses reveal distinct CD4 T cell subsets and functional differences in BS and STB. **(A)** UMAP visualisation of CD4 T cell subclusters from BS and STB samples (n = 7,787 cells after quality control). **(B)** Feature plots showing expression of canonical marker genes across eight CD4 subsets. **(C)** Proportional distribution of CD4 T cell subsets in BS and STB. **(D)** Ro/e enrichment analysis highlighting subset preference between BS and STB. **(E)** Venn diagram showing overlapping upregulated genes of IL-26-CD4 T cells between single-cell and bulk RNA-seq datasets. **(F)** PPI network of IL-26-CD4 T cell-associated DEGs. **(G)** Violin plots showing differential expression of CXCR3, IL2RG, IL23R, and IL-26 between BS and STB. **(H)** KEGG pathway enrichment analysis of IL-26- CD4 T cell-related genes. **(I)** Venn diagram showing overlapping upregulated genes of Cytotoxic_CD4T cells between single-cell and bulk RNA-seq datasets. **(J)** PPI network of Cytotoxic CD4 T cell–associated DEGs. **(K)** Violin plots showing differential expression of TNF, CD40LG, CD274, and LTA between BS and STB. **(L)** KEGG pathway enrichment analysis of Cytotoxic CD4 T cell–related genes. The symbol * indicates p < 0.05.

CD4 T cells were reclustered, and based on differential expression of canonical cell markers and characteristic genes, eight transcriptionally distinct subsets were identified: Naïve CD4 T, IL26-CD4 T, CAPG-CREM-Memory CD4 T, Follicular-Helper CD4 T, Effector-Memory CD4 T, Cytotoxic CD4T, Proliferating CD4 T, and Treg (**Figure 3A**). Feature plots of representative signature genes clearly delineated the molecular features of each subset (**Figure 3B**). Comparative analysis of subset distributions between BS and STB, combined with Ro/e enrichment, revealed distinct disease-associated profiles. IL26-CD4 T, Cytotoxic CD4 T, Proliferating CD4 T, and Treg subsets were enriched in STB. Conversely, Naïve CD4 T, CAPG-CREM-Memory CD4 T, Follicular-Helper CD4 T, and Effector-Memory CD4 T subsets were predominant in BS, reflecting a memory-biased T-cell phenotype characteristic of chronic bacterial infection (**Figures 3C, D**). These distribution patterns are consistent with sustained Th1-skewed granulomatous inflammation in tuberculosis, in contrast to the chronic low-grade inflammatory profile observed in brucellosis (Flynn and Chan, 2001; Martirosyan and Gorvel, 2013).

Further analysis of IL26-CD4 T cells in STB revealed only 24 overlapping genes between their differentially expressed genes and the upregulated genes identified by bulk RNA-seq (**Figure 3E**), indicating a highly specific transcriptomic signature unique to this subset. Protein–protein interaction (PPI) network analysis identified CXCR3, IL2RG, IL23R, and IL-26 as central nodes (**Figure 3F**), suggesting that this population functions as a critical hub integrating Th1 and Th17 immune responses. At the transcriptional level, IL26-CD4 T cells in STB exhibited higher expression levels of CXCR3, IL2RG, IL23R, and IL-26 compared with BS, reflecting a relatively enhanced pro-inflammatory potential (**Figure 3G**). KEGG pathway enrichment analysis revealed that these genes were mainly involved in cytokine-cytokine receptor interaction, NF-κB signalling, JAK-STAT signalling, Th17 differentiation, and chemokine signalling pathways (**Figure 3H**). The robust expansion and inflammatory pathway activation of IL26-CD4 T cells in STB highlight this subset as a key effector population driving chronic inflammation and tissue destruction in spinal tuberculosis.

Similarly, Cytotoxic CD4 T cells in STB displayed distinct transcriptional features, sharing only 28 overlapping genes between single-cell and bulk RNA-seq datasets (**Figure 3I**). PPI network analysis pinpointed TNF, CD40LG, CD274 (PD-L1), and LTA as central molecules, implying that these cells mediate both inflammatory amplification and immune regulation (**Figure 3J**). At the gene expression level, TNF, CD40LG, CD274, and LTA showed significantly higher expression levels in STB compared with BS, suggesting a dual functional profile combining pro-inflammatory activation and immunosuppressive potential (**Figure 3K**). KEGG enrichment analysis indicated predominant involvement in NF-κB and T-cell receptor (TCR) signalling, together with multiple autoimmune-related pathways (**Figure 3L**). Collectively, these findings demonstrate that Cytotoxic CD4 T cells co-express pro-inflammatory mediators (TNF, CD40LG) and inhibitory signals (PD-L1), encapsulating the immunopathological paradigm of STB, where inflammation-driven activation and immune evasion coexist.

### 3.4 Single-cell and bulk RNA-seq reveal differences in CD8 T cell subsets between BS and STB

**Figure 4:**
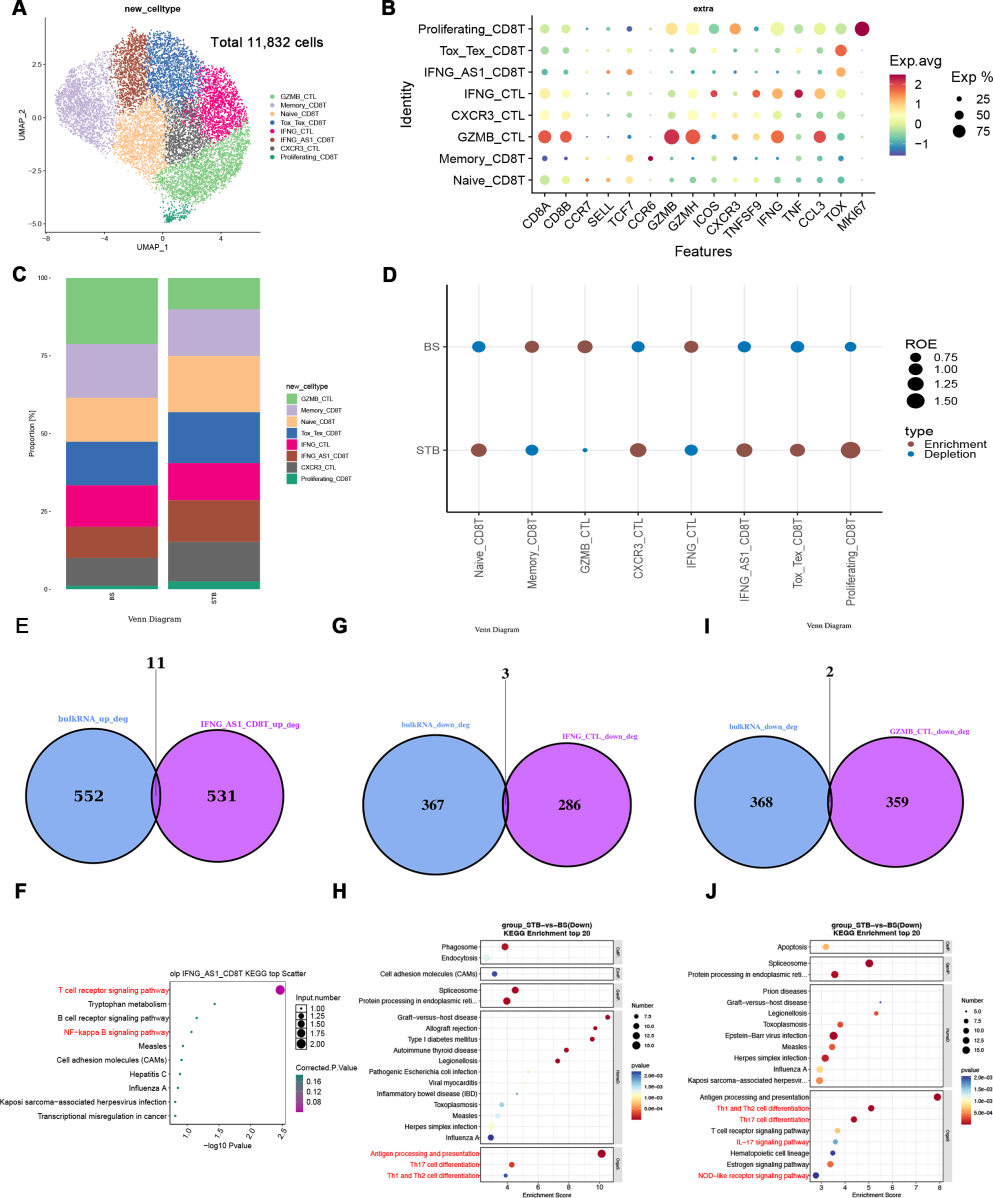
Single-cell and bulk RNA-seq analyses reveal distinct CD8 T cell subsets and functional differences between BS and STB. **(A)** UMAP visualisation of CD 8 T cell subclusters identified from BS and STB samples (n = 11,832 cells after quality control). **(B)** Feature plots showing canonical marker gene expression across eight CD8T cell subsets. **(C)** Proportional distribution of CD8 T cell subsets in BS and STB. **(D)** Ro/e enrichment analysis indicating preferential enrichment of specific subsets in BS and STB. **(E)** Venn diagram showing overlapping upregulated genes of IFNG-AS1-CD8 T cells between single-cell and bulk RNA-seq datasets. **(F)** KEGG pathway enrichment analysis of IFNG-AS1- CD8 T cell-associated DEGs. **(G)** Venn diagram showing overlapping downregulated genes of IFNG-CTL cells between single-cell and bulk RNA-seq datasets. **(H)** KEGG pathway enrichment analysis of IFNG-CTL-related genes. **(I)** Venn diagram showing overlapping downregulated genes of GZMB-CTL cells between single-cell and bulk RNA-seq datasets. **(J)** KEGG pathway enrichment analysis of GZMB-CTL-related genes.

CD8 T cells were reclustered to delineate transcriptional heterogeneity, and based on the differential expression of canonical markers and characteristic genes, eight distinct subsets were identified: GZMB-CTL, Memory CD8 T, Naïve CD8 T, Tox-Tex CD8 T, IFNG-CTL, IFNG-AS1-CD8 T, CXCR3-CTL, and Proliferating CD8 T (**Figure 4A**). Feature plots of representative marker genes clearly defined the molecular features distinguishing each subset (**Figure 4B**). Comparative distribution analysis combined with Ro/e enrichment revealed distinct enrichment patterns across disease groups. Specifically, Naïve CD8 T, CXCR3-CTL, IFNG-AS1-CD8 T, Tox-Tex-CD8 T, and Proliferating CD8 T subsets were predominantly enriched in STB, whereas GZMB-CTL, Memory CD8 T, and IFNG-CTL subsets were more abundant in BS (**Figures 4C, D**). These divergent enrichment profiles indicate distinct relative activation states and functional polarisation of CD8 T cells between the two conditions.

Further analysis of IFNG-AS1 CD8 T cells in STB revealed only 11 overlapping genes between their single-cell differentially expressed genes and the bulk RNA-seq dataset (**Figure 4E**), indicating a distinct transcriptional signature specific to this subset. KEGG pathway enrichment analysis demonstrated that these genes were primarily involved in T-cell receptor signalling, NF-κB signalling, B-cell receptor signalling, and cell adhesion molecule pathways (**Figure 4F**). Activation of the IDO-kynurenine metabolic axis has been implicated in T-cell suppression and immune regulation in tuberculosis lesions, potentially contributing to functional exhaustion under chronic antigen exposure (O’Garra et al., 2013).

In BS, the overlap between downregulated genes of IFNG-CTL and GZMB-CTL subsets at the single-cell level and bulk RNA-seq data was minimal (**Figures 4G, I**). In contrast, BS GZMB-CTL and IFNG-CTL subsets demonstrated preserved enrichment in antigen processing and presentation, TCR signalling, Th1/Th2/Th17 differentiation, and NOD-like receptor pathways (**Figures 4H, J**), consistent with prior single-cell and functional studies describing an integrated CTL-helper immune network in Brucella infection (Wang et al., 2024).

### 3.5 Th1 and Th17 responses and apoptosis features of T cells in BS and STB

To quantify functional states of T cells, AUCell-based gene set scoring was applied to curated Th1, Th17, exhaustion, apoptosis, and migration signatures (Yang et al., 2023).

**Figure 5:**
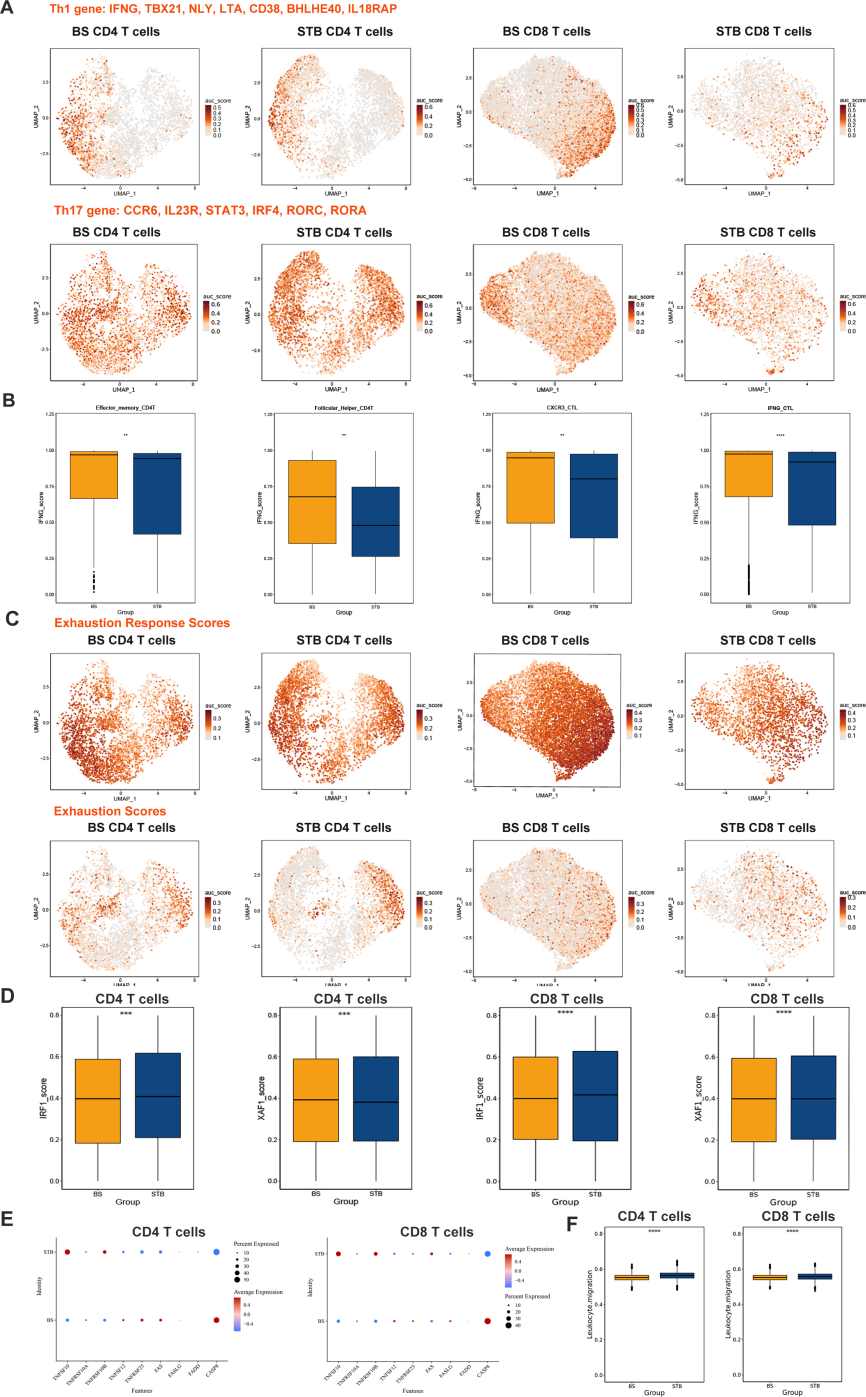
Th1/Th17 responses and apoptosis characteristics of T cells in BS and STB. **(A)** AUCell-based scoring of curated Th1 and Th17 gene sets in CD4 and CD8 T cells. **(B)** Box plots showing the IFNG expression in CD4 and CD8 T cells subset per condition. **(C)** AUCell-based scoring of curated exhaustion response scores and exhaustion scores gene sets in CD4 and CD8 T cells. **(D)** Box plots showing XAF1 and IRF1 expression in CD4 and CD8 T cells between BS and STB. **(E)** Dot plots showing the expression of selected apoptosis-associated genes in T cells between BS and STB. **(F)** Box plots of leukocyte migration scores in T cells between BS and STB. The symbols indicate statistical significance as follows: *p < 0.05; **p < 0.01; ***p < 0.001; ****p < 0.0001; ns, not significant.

Feature plots revealed that Th1 signatures were broadly enriched in both CD4 and CD8 T cells in BS and STB (**Figure 5A**). Notably, Th1 activity in STB was more evenly distributed across CD4 T cell subsets, whereas in BS it was more concentrated within CD8 T cells. Th17 activity was largely restricted to CD4 T cells and exhibited a more continuous activation pattern in STB compared with a more dispersed distribution in BS (**Figure 5A**). Quantitative analysis of AUCell scores further supported these observations. Th1 scores were significantly higher in both CD4 and CD8 T cells in STB compared with BS, whereas Th17 scores were significantly increased in CD4 T cells but showed no significant difference in CD8 T cells between the two groups (**Supplementary Figures 2A, B**). Consistent with these findings, IFNG activity scores (**Figure 5B**) were significantly higher in effector memory CD4 T cells and IFNG⁺ cytotoxic T lymphocytes (CTLs) in STB compared with BS, whereas relatively elevated IFNG scores were observed in Tfh cells and CXCR3⁺ CTLs in BS. Exhaustion-related signatures (**Figure 5C**) showed a broader distribution in BS, suggesting more widespread antigen-driven T cell modulation. Quantitative analysis demonstrated that overall exhaustion scores did not differ significantly between BS and STB in either CD4 or CD8 T cells. Further analysis revealed that exhaustion response scores were significantly higher in both CD4 and CD8 T cells in BS compared with STB, indicating that specific exhaustion-related functional programmes are more actively engaged in BS despite comparable overall exhaustion levels (**Supplementary Figures S2C, D**). Apoptosis-related and IFN-responsive transcriptional activity also differed between conditions. Quantitative scoring demonstrated that IRF1 and XAF1 signatures were significantly higher in STB compared with BS (**Figure 5D**). In agreement with this, gene-level analysis showed increased expression of extrinsic apoptosis pathway components, including TNFSF10 (TRAIL), FAS/FASLG, and CASP8, in STB T cells (**Figure 5E**). In addition, leukocyte migration scores were significantly higher in both CD4 and CD8 T cells in STB compared with BS (**Figure 5F**), indicating enhanced migratory potential.

Collectively, these results indicate that STB is characterised by enhanced Th1/Th17 activity, increased apoptosis-related signalling, and greater migratory capacity, whereas BS exhibits relatively restrained inflammatory activation. Although overall exhaustion levels were comparable, BS displayed significantly higher exhaustion-related response activity in both CD4 and CD8 T cells.

### 3.6 Quantitative evaluation of functional variations of T cells in BS and STB

**Figure 6:**
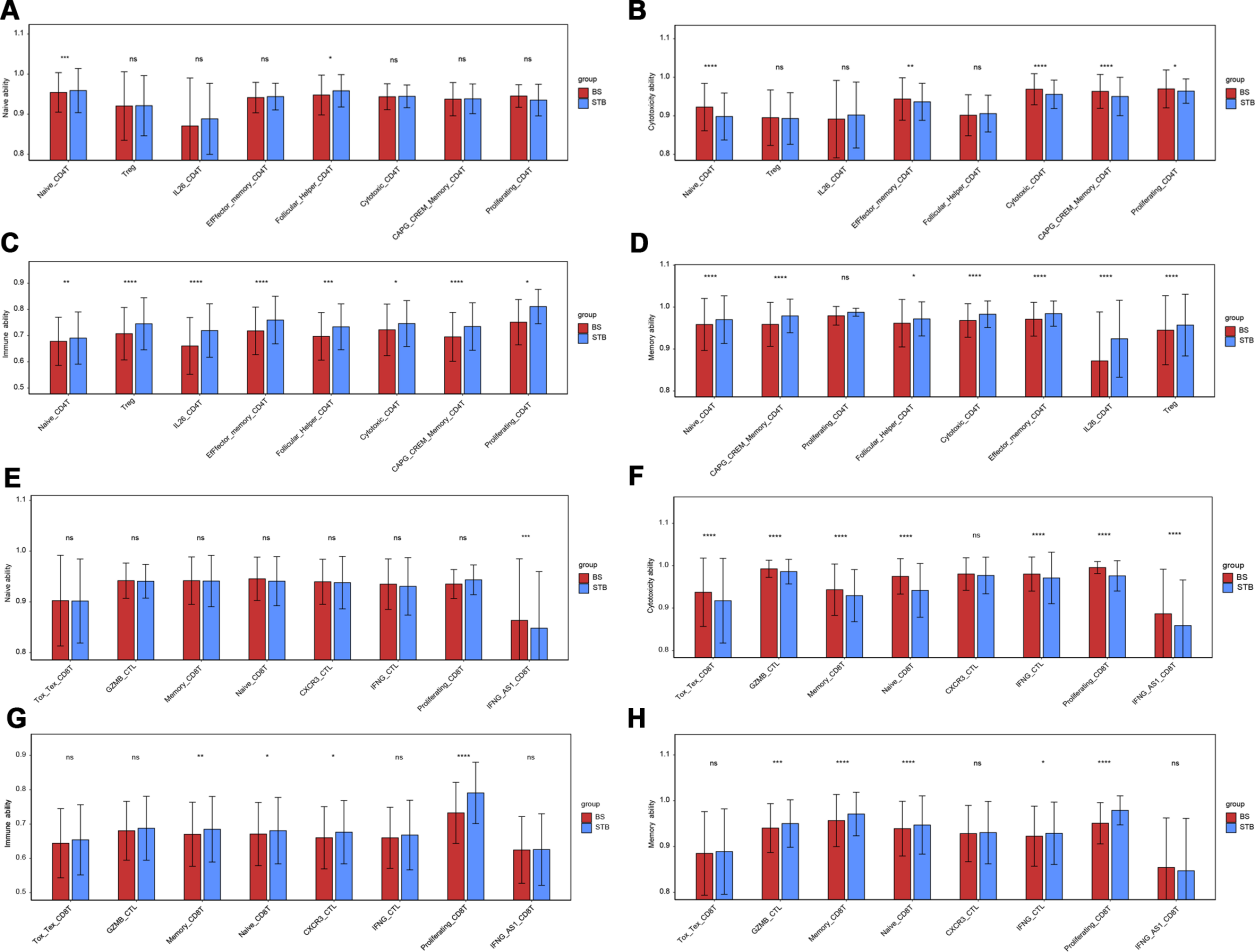
Quantitative evaluation of functional variations of T cells in BS and STB. **(A–D)** Functional of naive ability, cytotoxic ability, immune ability and memory ability scoring of CD4T cells subsets in BS and STB. **(E–H)** Functional of naive ability, cytotoxic ability, immune ability and memory ability scoring of CD 8T cells subsets in BS and STB. *p < 0.05; **p < 0.01; ***p < 0.001; ****p < 0.0001; ns, not significant.

We systematically compared the functional characteristics of CD4 and CD8 T cells subsets in BS and STB (**Figures 6A–H**). In the CD4 T cell compartment, STB patients exhibited a relatively higher proportion of Naïve CD4 T cells compared with BS (showing enhanced priming potential, cytotoxicity, and immune competence, *p* < 0.01), indicating that the local microenvironment in STB favours T cell activation and effector differentiation. Cytotoxic CD4 T and Follicular-Helper CD4 T subsets were also enriched, suggesting the coexistence of CD4 T cells mediated cytotoxicity and augmented B cells assistance through the Tfh axis. IL-26-CD4 T cells in STB displayed markedly elevated immune and memory potential. In tuberculosis, IL-26 upregulation has been shown to mediate direct mycobacterial killing and TLR2/9-dependent activation of antigen-presenting cells, thereby implicating this subset as a dual-function population involved in host defence and granuloma-associated inflammation (Meller et al., 2015). For CD8 T cells, GZMB-CTLs, IFNG-CTLs, and Proliferating CTLs showed higher abundance in STB compared with BS, consistent with enhanced cytotoxic capacity, immune competence, and memory potential. In BS, Naïve CD8T and Memory CD8T subsets were diminished, displaying attenuated effector functionality but elevated IFNG-AS1-CD8T expression, suggesting a state of chronic inflammation sustained by lncRNA-mediated regulation of IFN-γ (Petermann et al., 2019). IFN-γ signature scores were concordant with cytotoxic and memory-related pathways: STB T cells-particularly CD8 CTLs and selected CD4 subsets-exhibited higher IFN-γ activity, consistent with the Th1/IFN-γ–CXCR3 axis mediating tissue homing and protective responses (Shanmugasundaram et al., 2020). CXCR3-IFN-γ-T cells, responsive to CXCL9/10/11 chemokines, preferentially accumulate within pulmonary and granulomatous regions, where their abundance inversely correlates with bacterial load.

### 3.7 Differentiation trajectories and representative gene expression patterns of T cells in BS and STB

**Figure 7:**
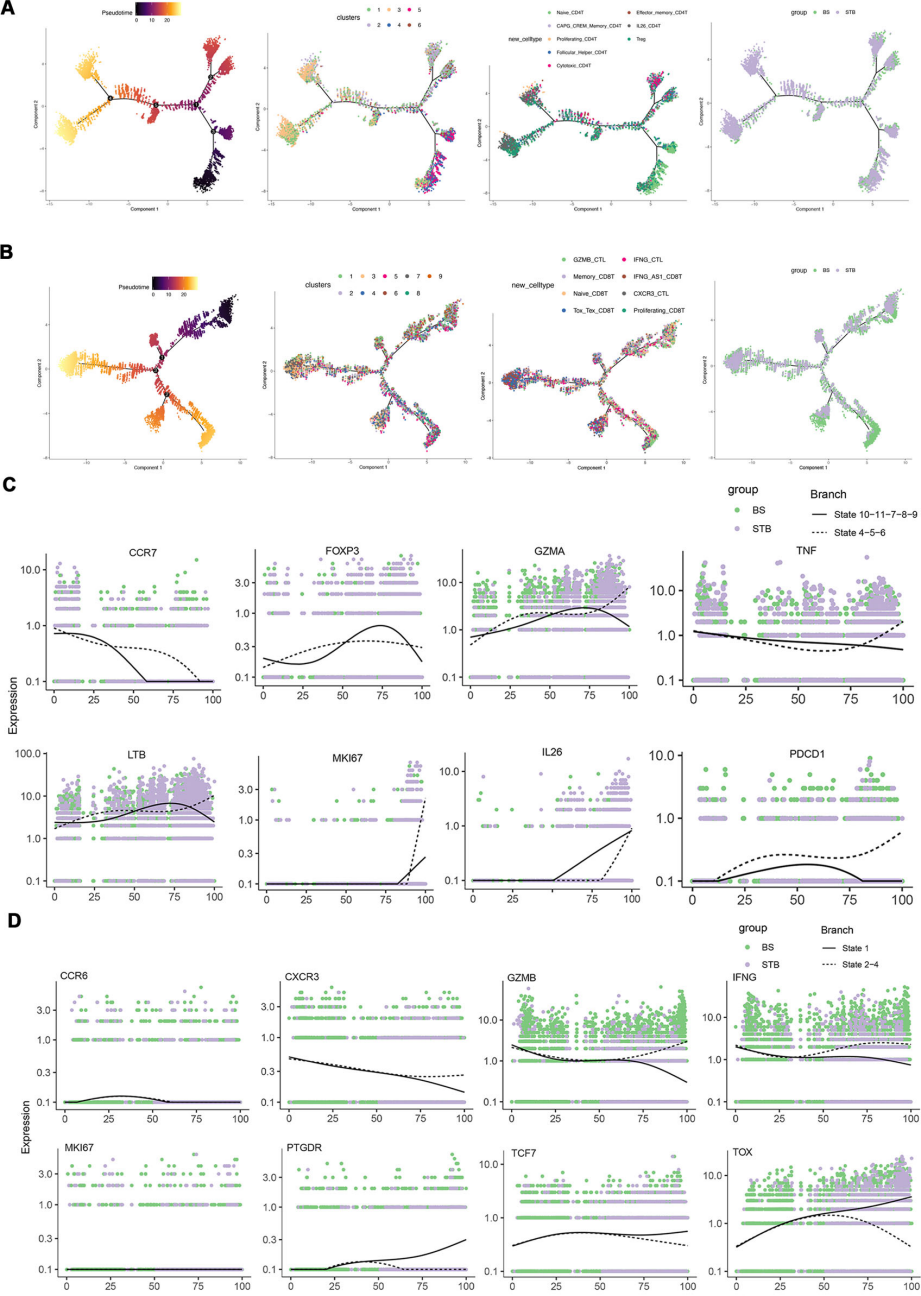
Differentiation trajectories and representative gene expression patterns of T cells in BS and STB. **(A, B)** Pseudotime trajectories of CD4 and CD8 T cell subsets, coloured by differentiation state, cell subtype, pseudotime, and group, respectively. **(C)** Expression dynamics of representative genes-CCR7, GZMA, FOXP3, TNF, MKI67, LTB, IL26 and PDCD1 in CD4 T cells, along pseudotime trajectories. **(D)** Expression dynamics of representative genes-CCR6, CXCR3, GZMB, IFNG, MKI67, PTGDR, TCF7 and TOX in CD8 T cells, along pseudotime trajectories.

To investigate functional alterations in T cells between BS and STB, pseudotime trajectory analysis was performed on CD4 and CD8 T cells subsets. In trajectory reconstruction, CD4 T cells in both cohorts originated from Naïve_CD4T populations and subsequently diverged into multiple terminal branches (**Figure 7A**). However, the differentiation trajectories exhibited distinct patterns between the two groups. In BS patients, CD4 T cells were predominantly retained at intermediate states, enriched within CAPG-CREM-Memory CD4 T, Tfh, and Treg subsets. Pseudotime dynamics demonstrated a gradual increase in FOXP3 expression during late stages, accompanied by a slower decline in CCR7 (**Figure 7C**), suggesting partial differentiation arrest with a bias towards regulatory or memory-like phenotypes. In contrast, CD4 T cells in STB were preferentially committed to robust effector branches, including Effector Memory CD4T and IL26-CD4 T subsets (**Figure 7A**). At the terminal pseudotime, expression of effector and proliferative genes such as GZMA, IFNG, and MKI67 continued to rise (**Figure 7C**), indicating differentiation towards highly activated Th1 and CTL lineages.

CD8 T cells in both groups originated from Naïve CD8 T and Memory CD8 T populations and subsequently diverged into multiple terminal branches, exhibiting pronounced heterogeneity (**Figure 7B**). In BS patients, CD8T cells primarily differentiated into GZMB-CTL and IFNG-CTL lineages, with pseudotime dynamics revealing progressive upregulation of cytotoxic and effector genes, including GZMA, IFNG, and TNF (**Figure 7C**). Conversely, in STB patients, CD8 T cells were skewed towards Tox-Tex-CD8 T (exhaustion-like) and Proliferating CD8 T trajectories (**Figure 7B**). At the terminal pseudotime, PDCD1 (PD-1) and TOX expression remained persistently elevated, accompanied by increased MKI67 and IL26 expression (**Figure 7D**). In summary, CD8 T cells in BS were predominantly directed towards effector cytotoxic fates, whereas those in STB displayed concurrent features of exhaustion and sustained proliferation, reflecting fundamentally distinct mechanisms of immune regulation and disease progression.

Collectively, these findings indicate that CD4 T cells in STB are skewed toward terminal inflammatory effector fates, whereas BS CD4 T cells remain preferentially in intermediate or regulatory states. In parallel, BS CD8 T cells predominantly adopt cytotoxic effector trajectories, while STB CD8 T cells exhibit coupled proliferation and exhaustion features, highlighting fundamentally distinct immune differentiation programmes induced by the two pathogens.

### 3.8 Distinct transcriptional regulatory programs of CD8 and CD4 T cells in BS and STB

To characterise transcriptional regulatory landscapes, regulon activity analysis was performed across major CD4 and CD8 T cell subsets.

**Figure 8:**
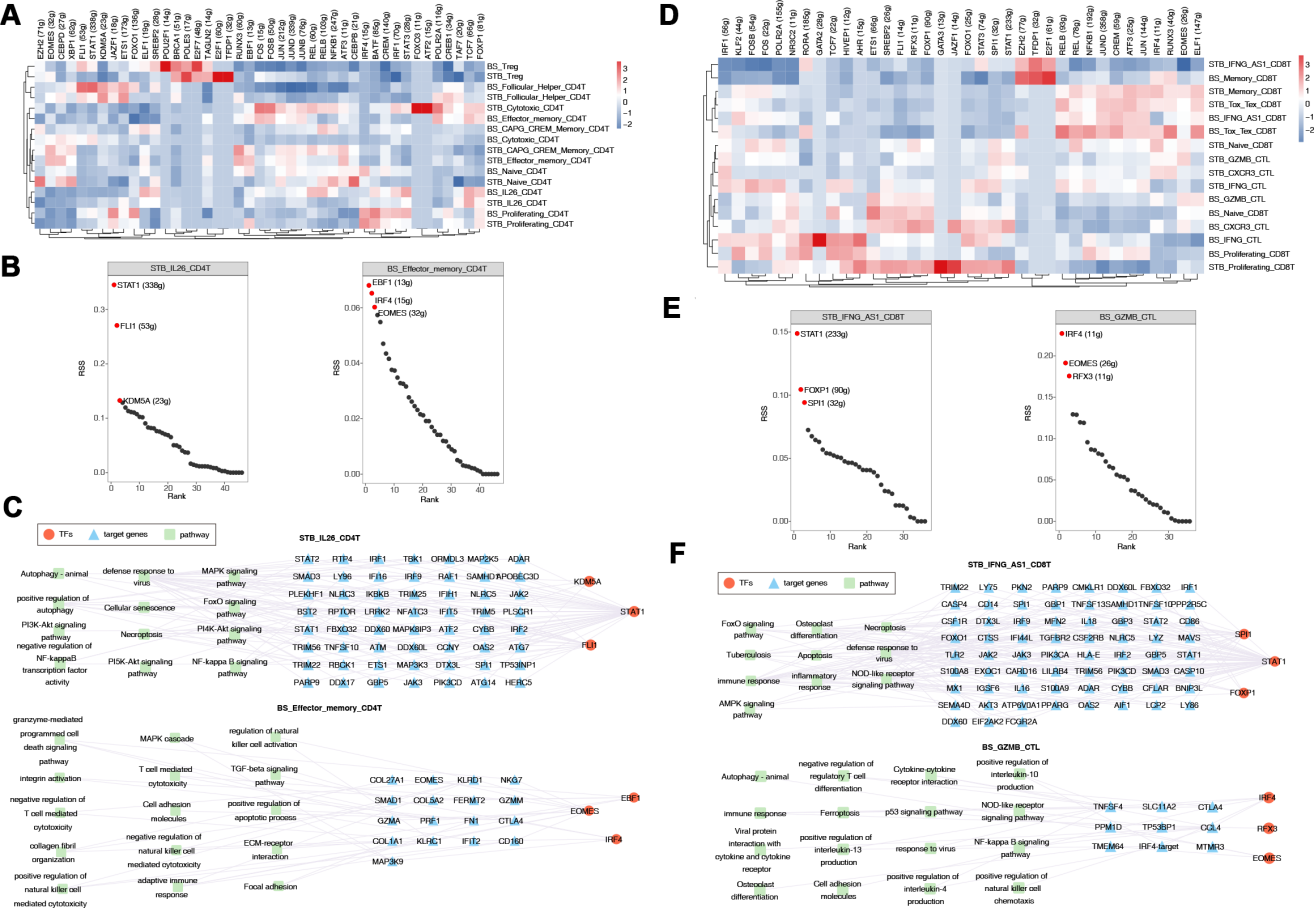
Distinct transcriptional regulatory programmes of CD 4 and CD 8 T cells in BS and STB. **(A)** Heatmap of regulon activity scores (RAS) across CD4 T cell subsets in BS and STB. **(B)** Representative regulons specifically activated in IL26- CD4 T cells from STB (left) and Effector-memory CD4 T cells from BS (right). **(C)** Transcription factor-target gene-pathway networks of STB IL26-CD4T (up) and BS Effector-memory CD4T (down). **(D)** Heatmap of regulon activity scores (RAS) across CD8 T cell subsets in BS and STB. **(E)** Representative regulons specifically activated in STB IFNG-AS1-CD8T cells (left) and BS GZMB-CTL cells (right). **(F)** Transcription factor-target gene-pathway networks of STB IFN-AS1-CD8T (up) and BS GZMB-CTL (down).

In CD4 T cells, marked divergence was observed between STB and BS (**Figure 8A**). STB IL26-CD4T cells showed strong activation of STAT1, FLI1, and KDM5A regulons, with high regulon specificity scores (STAT1, FLI1, KDM5A) (**Figure 8B**). In contrast, BS Effector-Memory CD4T cells preferentially activated IRF4 and EOMES regulons. Transcription factor-target gene network analysis further demonstrated that STB IL26-CD4T cells were enriched in interferon-stimulated gene modules (IFI16, IFI44L, OAS2, GBP family, TRIM family, DDX60), associated with inflammatory and cell death-related pathways (**Figure 8C**). Conversely, BS Effector-Memory CD4T cells displayed coordinated regulation of cytotoxic genes (PRF1, GZMA, GZMM, NKG7) together with regulatory and adhesion-related genes (CTLA4, SMAD1, COL, FN1, FERMT2). In CD8 T cells, STB IFNG-AS1-CD8T subsets exhibited dominant activation of STAT1, IRF family members (IRF1/7/9), FOXP1, and SPI1 regulons, with high specificity scores (STAT1: 233 genes; FOXP1: 90 genes; SPI1: 32 genes) (**Figures 8D, E**). Target genes were enriched in interferon signalling, NOD-like receptor pathways, autophagy, necroptosis, and inflammatory responses (**Figure 8F**). In contrast, BS GZMB-CTL cells preferentially activated IRF4, EOMES, and RFX3 regulons, regulating cytotoxic genes (PRF1, GZMB, GZMM, NKG7) and immune regulatory modules (TNFSF4, CCL4, CTLA4, TP53BP1). Enriched pathways included NF-κB, p53 signalling, ferroptosis, autophagy, and cytokine-receptor interactions. Despite these divergent transcriptional programmes, both diseases showed enrichment of osteoclast differentiation pathways.

### 3.9 Cell-cell communication landscapes in BS and STB

**Figure 9:**
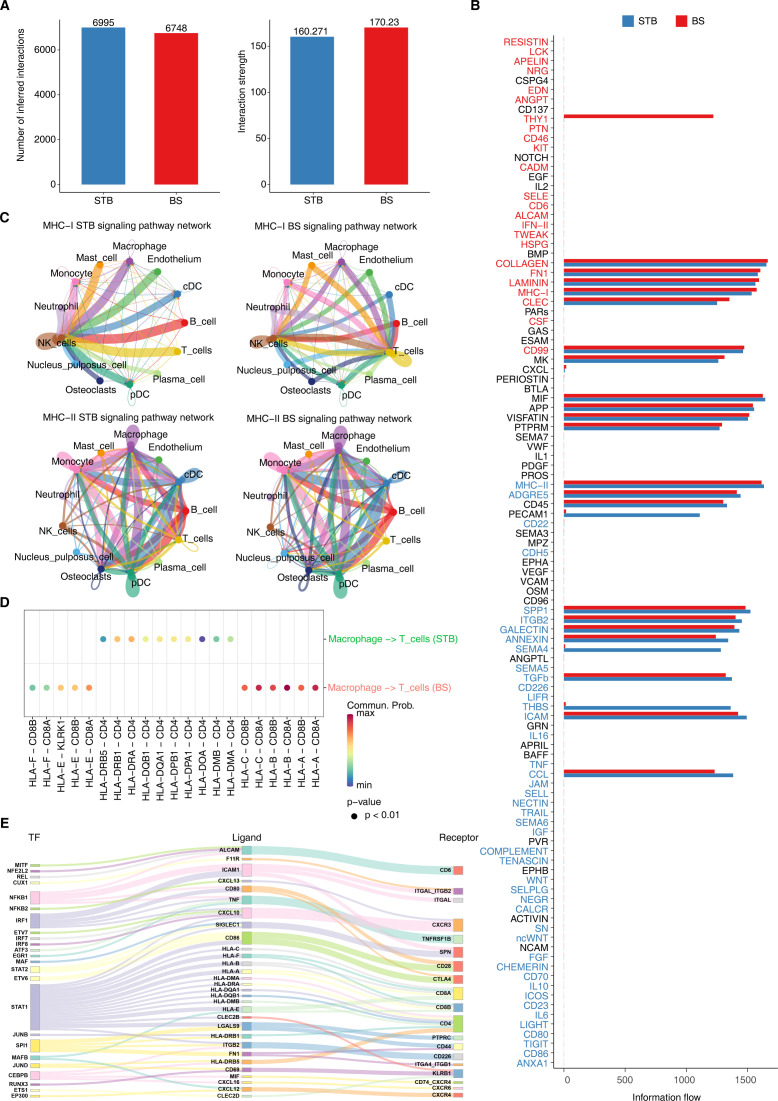
Comparative cell–cell communication landscapes in BS and STB. **(A)** Overall numbers and strengths of intercellular communications among major cell types in BS and STB. **(B)** Overview of enriched intercellular signalling pathways in BS and STB. **(C)** Network diagrams showing cell–cell interactions mediated by MHC-I and MHC-II signalling in BS and STB. **(D)** Bubble plots depicting the relative expression of ligand–receptor pairs involved in MHC-I and MHC-II signalling pathways. **(E)** Sankey diagram illustrating the transcription factor–ligand–receptor network (transcription factors and ligands derived from macrophages and receptors from T cells).

STB exhibited a slightly higher number of ligand–receptor interactions than BS (6995 vs. 6748), whereas BS displayed greater overall interaction strength (170.23 vs. 160.27) (**Figure 9A**), indicating a communication pattern characterised by fewer but more focused interactions. At the pathway level (**Figure 9B**), BS demonstrated enrichment of MHC-I, CD137, ICAM/VCAM–integrin, and extracellular matrix (ECM) signalling pathways, consistent with stabilised cytotoxic T lymphocyte (CTL) synapses and reinforced cytotoxic function. In contrast, STB showed higher activity in MHC-II, CD80/CD86, ICOS, TIGIT, IL-10, and CHEMERIN pathways, reflecting a Th1-dominant yet immunoregulatory communication network. Within the MHC-I signalling context (**Figure 9C**), STB networks were organised around NK-T cell interactions with macrophages serving as central hubs, suggesting innate immune–driven T cell activation. In BS, B cell-conventional dendritic cell (cDC) connectivity was markedly enhanced, underscoring a more prominent contribution of B cells to antigen presentation. In MHC-II signalling (**Figure 9C**), STB exhibited a dense and evenly distributed network comprising macrophages, cDCs, B cells, and T cells, whereas BS displayed thicker B cell-cDC/pDC connections but reduced NK involvement, again highlighting B cell and dendritic cell dominance in antigen presentation and immune modulation. Ligand–receptor binding analysis (**Figure 9D**) revealed pathogen-specific interaction preferences. In STB, HLA-DR/DQ/DP-CD4 binding predominated, supporting an MHC-II-CD4 signalling axis that drives Th1 activation and granuloma formation. Conversely, BS favoured HLA-A/B/C/E-CD8A/B interactions, consistent with an MHC-I-CD8 axis promoting cytotoxic T lymphocyte (CTL) activation. Both groups exhibited HLA gene induction mediated by IFN-γ-STAT1/IRF signalling (**Figure 9E****).** However, STB additionally engaged the NF-κB-CXCL10/TNF axis, establishing a broad Th1-inflammatory programme, whereas BS combined adhesion and integrin pathways with CD137 co-stimulatory signalling, generating a high-intensity CTL microenvironment.

Overall, STB was biased towards the MHC-II-CD4 axis, emphasising Th1 helper responses and macrophage activation, while BS was oriented towards the MHC-I-CD8 axis, reinforcing CTL-mediated cytotoxicity. These distinctions highlight tuberculosis as an innate-Th1-driven, diffusely inflammatory state, whereas brucellosis exhibits a more focused CTL pathway supported by B cell and dendritic cell cooperation, providing a mechanistic rationale for pathogen-specific immunomodulatory strategies.

## 4 Discussion

In this study, we integrated single-cell with bulk RNA sequencing, to comparatively and systematically delineate the T cell immune landscape within intervertebral disc tissues infected by STB and BS. Our analyses revealed striking differences between these two chronic spinal infections in terms of CD4 and CD8 T cells subset composition, functional states, differentiation trajectories, transcriptional regulatory networks, and cell–cell communication patterns. Bulk RNA sequencing provides an averaged transcriptional profile across heterogeneous cell populations and therefore has lower resolution than single-cell RNA sequencing. The apparent differences between the two platforms in this study are primarily attributable to this averaging effect rather than true biological inconsistency. Nevertheless, our integrative analyses demonstrate that bulk RNA-seq retains major cell-type-associated transcriptional signals at the tissue level, supporting its role as a complementary dataset to validate overall transcriptional patterns, while single-cell RNA-seq provides the primary evidence for cell-type-specific mechanisms.

In STB, specific subsets including IL26-CD4 T cells, Cytotoxic CD4 T cells, and IFNG-AS1-CD8 T cells-were markedly expanded, all exhibiting strong activation of STAT1/IRF regulons alongside broad upregulation of interferon-stimulated genes (ISGs). This pattern is highly consistent with the systemic “interferon signature” previously reported in tuberculosis patients (Berry et al., 2010). IL26-CD4 T cells displayed activation of the STAT1/FLI1/KDM5A regulatory network and enrichment in JAK-STAT, NF-κB, and Th17 signalling pathways. Functionally, IL26 has been shown to enhance macrophage bactericidal activity and induce antimicrobial peptides in tuberculosis; however, sustained IL26 elevation may also amplify inflammation and tissue injury, reflecting its characteristic “double-edged sword” effect (Hawerkamp et al., 2020).

Cytotoxic CD4 T cells upregulated TNF and CD40LG (pro-inflammatory and cytotoxic mediators) together with CD274 (PD-L1) (an immunosuppressive molecule), indicating a complex immune landscape in which pro-inflammatory activation coexists with inhibitory feedback regulation. The IFNG-AS1-CD8 T subset, a key population in STB, was transcriptionally regulated by the STAT1/FOXP1/SPI1 axis and controlled a broad range of ISGs-ncluding TRIM22, MX1, OAS2, GBP, and IFI44L-nriched in tuberculosis, NOD-like receptor, autophagy, necroptosis, and osteoclast differentiation pathways. This profile reflects a state of sustained antigen-driven inflammation, immune exhaustion, and aberrant proliferation, consistent with the functional exhaustion phenotype observed in TB lesions (Pan et al., 2023). Furthermore, the prominent enrichment of tryptophan metabolism in STB suggests that the IDO-ynurenine-hR immunometabolic axis may exert a pivotal role in modulating T cell function, providing a “metabolic brake” within a hyperinflammatory milieu (Sia and Rengarajan, 2019). This mechanism may explain why STB T cells, despite producing high levels of IFN-γ, are prone to exhaustion and functional impairment.

In contrast to STB, patients with BS exhibited marked expansion of Effector-memory CD4 T cells, GZMB-CTL, and IFNG-CTL subsets, whose regulatory networks were centred on IRF4, EOMES, and RFX3. IRF4 and EOMES are canonical transcriptional drivers of effector CTL differentiation and cytotoxic programmes, promoting the expression of PRF1, GZMB, and IFNG (Cruz-Guilloty et al., 2009; Raczkowski et al., 2013). RFX3, an immune-associated transcription factor involved in homeostatic regulation, has been shown to modulate T cell proliferation and stability through the miR-150 axis (Chirichella et al., 2022). Pathway enrichment analysis revealed that BS CTLs were concurrently linked to cytotoxic effectors (PRF1, GZMA, GZMM) and immunoregulatory factors (CTLA4, CCL4, TP53BP1), encompassing NF-κB, p53, ferroptosis, autophagy, and IL-10/IL-4/IL-13 signalling pathways. This configuration reflects a dual transcriptional programme of “potent cytotoxicity coupled with concurrent homeostatic regulation”. Such an immune signature aligns closely with established immunopathological features of Brucella infection: protective immunity depends on Th1/IFN-γ-riven responses (Vitry et al., 2012), whereas its low-endotoxic lipopolysaccharide (LPS) and VirB type IV secretion system (T4SS) act to suppress excessive inflammation (Conde-Álvarez et al., 2012; Ke et al., 2015). Clinical studies have likewise reported increased Treg frequencies and elevated IL-10 levels in the peripheral blood of patients with brucellosis, corroborating our single-cell findings of a local immune milieu characterised by the coexistence of effector activation and regulatory restraint (Wang et al., 2024).

Both infections were characterised by Th1-dominated immune responses, yet they exhibited marked divergence in detail. In STB, CD4 Th1 and Th17 programmes were broadly distributed, accompanied by higher IFN-γ scores and increased IRF1/XAF1-mediated apoptotic stress, indicating a vigorous inflammatory response coupled with enhanced cell death. In contrast, BS demonstrated more focused CD8 Th1 activity, while elevated IFN-γ scores within Tfh and CXCR3-CTL populations reflected a coordinated interplay between Th1 and humoral immunity. Moreover, CD8 T cells in BS displayed higher Exhaustion Response scores, consistent with findings from chronic brucellosis models in which Brucella melitensis infection induces CD8 T cell exhaustion characterised by PD-1 and LAG-3 upregulation alongside reduced IFN-γ production (Durward-Diioia et al., 2015; Pellegrini et al., 2025a). Conversely, CD8 T cells in STB retained stronger effector functionality, albeit under sustained IFN-driven stress, underscoring a distinct balance between inflammatory activation and functional endurance.

Importantly, our findings extend the conceptual framework proposed by Lai et al (Lai et al., 2024). in pulmonary tuberculosis, in which infection outcomes are determined by the interactions between T-cell subsets and distinct macrophage niches. Although our human scRNA-seq dataset from spinal lesions did not directly label pathogen-positive host cells and therefore cannot unequivocally distinguish infected from bystander myeloid cells, our cell–cell communication analysis provides strong evidence supporting the presence of analogous T-cell-dependent regulation in STB. Specifically, these interaction patterns suggest that the dominant myeloid compartment in STB is embedded within a macrophage-centred, CD4 T cell-driven helper circuit that promotes inflammatory activation while simultaneously incorporating compensatory inhibitory feedback mechanisms, consistent with established models of Th1-mediated macrophage activation in tuberculosis (Berry et al., 2010; Hawerkamp et al., 2020). This configuration reflects a broad, Th1-dominated inflammatory landscape counterbalanced by regulatory feedback mechanisms, in which macrophages act as central intermediaries linking innate and adaptive immune responses (Sia and Rengarajan, 2019). In contrast, BS exhibited fewer but more intense communication events, dominated by the MHC-I-CD8 axis, together with ICAM/VCAM-integrin and CD137 signalling, forming a concentrated CTL-APC synaptic architecture, consistent with classical cytotoxic T cell immune synapse formation (Cruz-Guilloty et al., 2009). These findings suggest that, compared with the CD4⁺ T cell-macrophage regulatory network in STB, lesion-associated antigen-presenting or infected cells in BS preferentially engage cytotoxic CD8⁺ T-cell programmes. Consistent with this, studies of immunosuppressive mechanisms in Brucella infection have shown that PD-L1⁺ cells can secrete IL-1RA to inhibit CD4 and CD8 T cell responses, providing a mechanistic explanation for the attenuated T cell activity observed in BS (Pellegrini et al., 2025b). Taken together, these findings indicate that both STB and BS exhibit T-cell-mediated regulation of lesion-resident myeloid cells; however, the dominant immune synapse differs substantially, with STB biased towards macrophage-centred MHC-II–CD4 inflammatory regulation and BS towards MHC-I–CD8-driven cytotoxic immune responses.

This study has several limitations. First, pathogen-positive host cells were not directly labelled, preventing definitive distinction between infected and bystander myeloid populations; thus, our conclusions rely on inferred cell–cell communication patterns. Second, the absence of a normal nucleus pulposus control restricts the analysis to a direct comparison between STB and BS, such that the findings reflect relative differences between the two conditions and may not capture transcriptional alterations shared by both. In addition, RNA sequencing measures relative transcript abundance, which limits interpretation of absolute expression changes. Third, the relatively small sample size may not fully capture inter-individual heterogeneity. Future studies integrating spatial transcriptomics, host–pathogen dual sequencing, and larger cohorts will further refine these findings.

## 5 Conclusion

Within a comparative transcriptomic framework, this study delineates pathogen-specific T cell immunological programmes in STB and BS through integrated single-cell and bulk transcriptomic analyses. STB was characterised by IFN-STAT1-driven inflammation coupled with T cell exhaustion, featuring expanded IL26-CD4 and IFNG-AS1-CD8 subsets with sustained interferon signalling and metabolic stress. In contrast, BS was dominated by an IRF4/EOMES/RFX3-regulated effector–memory cytotoxic network that balanced potent killing activity with immune homeostasis. Despite these mechanistic divergences, both infections converged upon osteoclast differentiation and bone remodelling pathways, indicating that immune–bone metabolic coupling represents a common pathological endpoint leading to vertebral destruction. Collectively, these findings advance understanding of the immunopathology of spinal infections and identify potential targets for pathogen-specific, host-directed therapeutic interventions.

## Data Availability

The datasets have been deposited in the Genome Sequence Archive for Human (GSA-Human) under accession numbers HRA017497 and HRA017547.The records are publicly searchable. As the study contains human sequencing data, access is provided through controlled access in accordance with ethical and privacy regulations.

## Supplementary material

Supplementary Figure S1 Integration of bulk and single-cell RNA sequencing analyses. **(A)** Cell-type composition inferred from bulk RNA-seq using deconvolution analysis, showing the relative abundance of major cell populations across BS and STB samples. **(B)** Heatmap of representative marker genes for major cell types identified by single-cell RNA-seq, illustrating their relative expression patterns in bulk RNA-seq data. **(C)** Distribution of Pearson correlation coefficients between bulk RNA-seq profiles and single-cell-derived expression signatures, demonstrating overall concordance between the two datasets.

Supplementary Figure S2 Quantitative analysis of Th1/Th17 activity and exhaustion-related signatures in T cells. **(A–B)** Th1 and Th17 scores in CD4⁺ and CD8⁺ T cells. Th1 activity is increased in STB, while Th17 activity is elevated in CD4⁺ T cells only. **(C–D)** Exhaustion and exhaustion response scores in CD4⁺ and CD8⁺ T cells. Overall exhaustion shows no difference, whereas exhaustion response is increased in STB. Scores were calculated using AUCell and compared by Wilcoxon rank-sum test. ns, not significant; ***p* < 0.01; *****p* < 0.0001.
