# Supplementary figures and images for: Integration of single-cell and bulk RNA sequencing reveals divergent T-cell immune circuits and a shared osteo-immune pathway in spinal tuberculosis and brucellar spondylitis
Source: Front Cell Infect Microbiol. 2026 May 19;16:1811708. doi: 10.3389/fcimb.2026.1811708 (PMC13226540; doi:10.3389/fcimb.2026.1811708)

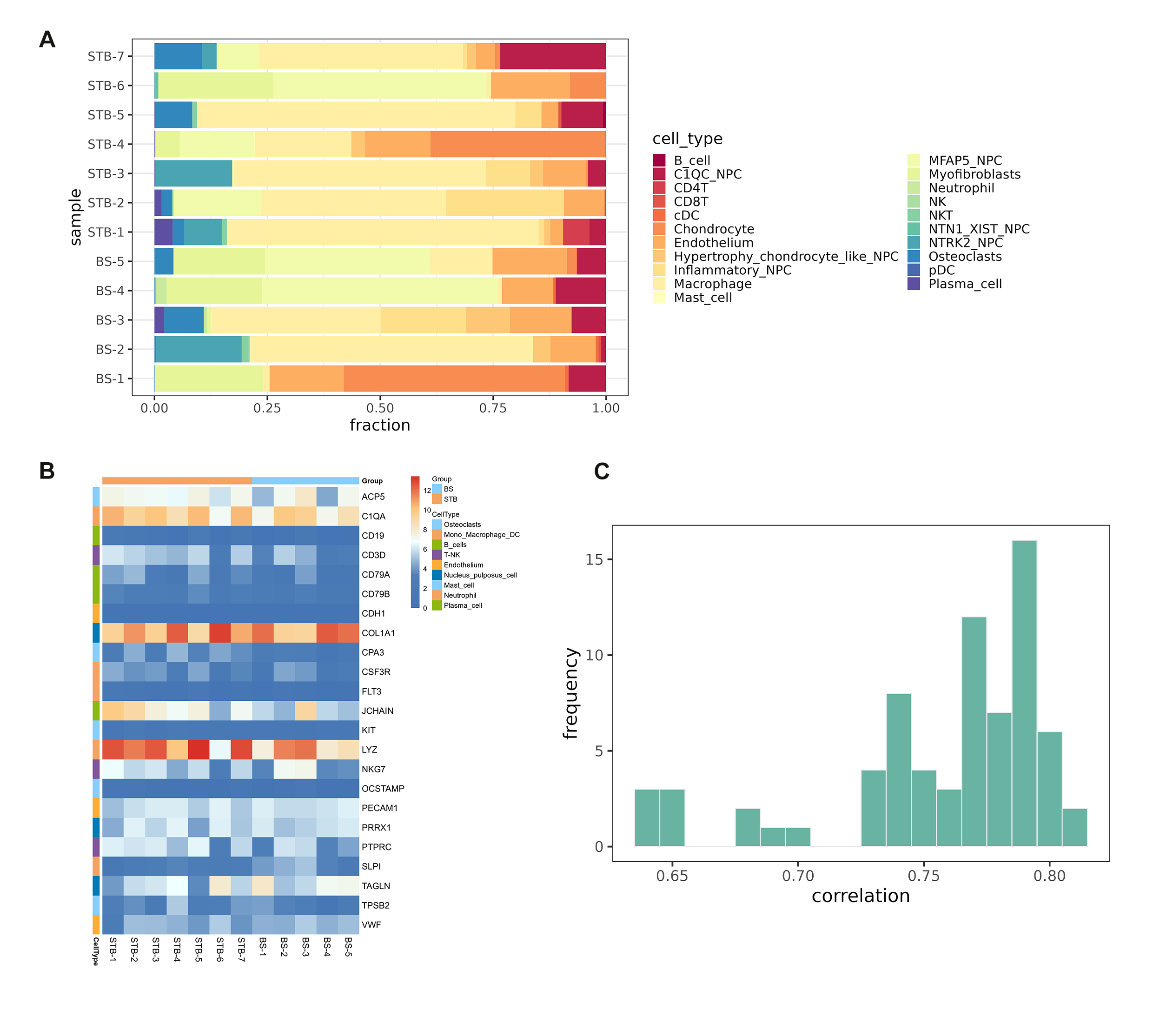

Supplement: Supplementary Figure S1 — Integration of bulk and single-cell RNA sequencing analyses. (A) Cell-type composition inferred from bulk RNA-seq using deconvolution analysis, showing the relative abundance of major cell populations across BS and STB samples. (B) Heatmap of representative marker genes for major cell types identified by single-cell RNA-seq, illustrating their relative expression patterns in bulk RNA-seq data. (C) Distribution of Pearson correlation coefficients between bulk RNA-seq profiles and single-cell-derived expression signatures, demonstrating overall concordance between the two datasets. [file Image1.tif]

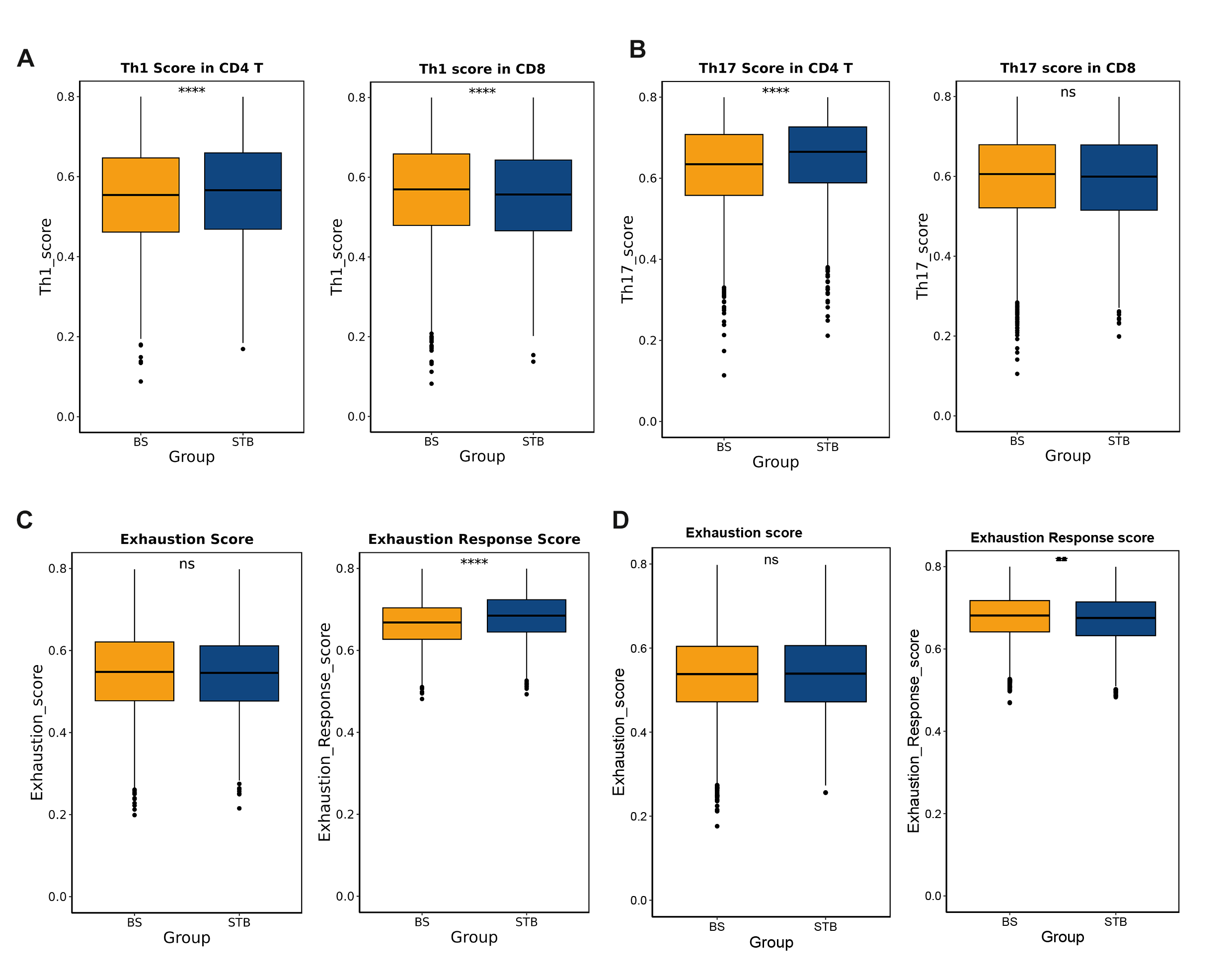

Supplement: Supplementary Figure S2 — Quantitative analysis of Th1/Th17 activity and exhaustion-related signatures in T cells. (A–B) Th1 and Th17 scores in CD4⁺ and CD8⁺ T cells. Th1 activity is increased in STB, while Th17 activity is elevated in CD4⁺ T cells only. (C–D) Exhaustion and exhaustion response scores in CD4⁺ and CD8⁺ T cells. Overall exhaustion shows no difference, whereas exhaustion response is increased in STB. Scores were calculated using AUCell and compared by Wilcoxon rank-sum test. ns, not significant; **p < 0.01; ****p < 0.0001. [file Image2.tif]
